# Heterologous expression and optimization using experimental designs allowed highly efficient production of the PHY US417 phytase in *Bacillus subtilis *168

**DOI:** 10.1186/2191-0855-2-10

**Published:** 2012-01-26

**Authors:** Ameny Farhat-Khemakhem, Mounira Ben Farhat, Ines Boukhris, Wacim Bejar, Kameleddine Bouchaala, Radhouane Kammoun, Emmanuelle Maguin, Samir Bejar, Hichem Chouayekh

**Affiliations:** 1 Laboratoire de Microorganismes et de Biomolécules, Centre de Biotechnologie de Sfax, Université de Sfax, Route de Sidi Mansour Km 6, BP "1177" 3018 Sfax, Tunisie; 2 INRA, UMR1319 Micalis, F-78350 Jouy en Josas, France; AgroParisTech, UMR Micalis, F-78350 Jouy en Josas, France

**Keywords:** Phytase, overexpression, *Bacillus subtilis*, multimeric DNA forms, experimental designs, thermostability

## Abstract

To attempt cost-effective production of US417 phytase in *Bacillus subtilis*, we developed an efficient system for its large-scale production in the generally recognized as safe microorganism *B. subtilis *168. Hence, the *phy *US417 corresponding gene was cloned in the pMSP3535 vector, and for the first time for a plasmid carrying the pAMβ1 replication origin, multimeric forms of the resulting plasmid were used to transform naturally competent *B. subtilis *168 cells. Subsequently, a sequential optimization strategy based on Plackett-Burman and Box-Behnken experimental designs was applied to enhance phytase production by the recombinant *Bacillus*. The maximum phytase activity of 47 U ml^-1 ^was reached in the presence of 12.5 g l^-1 ^of yeast extract and 15 g l^-1 ^of ammonium sulphate with shaking at 300 rpm. This is 73 fold higher than the activity produced by the native US417 strain before optimization. Characterization of the produced recombinant phytase has revealed that the enzyme exhibited improved thermostability compared to the wild type PHY US417 phytase strengthening its potential for application as feed supplement. Together, our findings strongly suggest that the strategy herein developed combining heterologous expression using a cloning vector carrying the pAMβ1 replication origin and experimental designs optimization can be generalized for recombinant proteins production in *Bacillus*.

## Introduction

Phytate/phytic acid (myo-inositol 1,2,3,4,5,6-hexakisphosphate; IP6) is the major storage form of phosphorus (P) in cereals, legumes and oilseeds accounting for ~60-90% of the total P content in plants (Rao et al. 2009). It is considered as an anti-nutrient factor since it forms insoluble complexes with nutritionally important ions such as Ca^2+^, Zn^2+^, Mg^2+^, Fe^2+^, and Mn^2+^. Phytases catalyze the release of phosphate from phytate, thereby generating less-phosphorylated myo-inositol derivatives (Li et al. 2010;Rao et al. 2009). Monogastric animals, such as poultry, swine and fish, cannot utilize phytate-P because their gastrointestinal tracts are deficient in phytase activity (Baruah et al. 2005). Supplementation of feeds destined to these animals with inorganic P is not only expensive, but also potentially polluting and non-sustainable. Indeed, in areas of extensive animal production, the supplementation of animal feed with inorganic P has led to increased manure P excretion levels and high soil P concentrations causing non-point pollution to surface and ground waters (Boesch et al. 2001). During the last two decades, exogenous phytases have been used as feed additives for monogastrics. Their inclusion into P-deficient diets is associated with substantial increases in total tract degradation of phytate-P and thus in the improvement of P bioavailability and growth performances (Li et al. 2010;Rao et al. 2009). Phytase also helps in the enhancement of vital minerals, amino acids and dietary carotenoids availability. Phytases are thus viewed as environmental-friendly products, which can reduce manure P excretion in intensive livestock management areas by limiting addition of exogenous P (Emiola et al. 2009;Jendza and Adeola 2009).

Although most of the commercially available phytases are fungal histidine acid phytases derived from *Aspergillus *species, bacterial phytases from the genus *Bacillus *are an alternative because of their high natural thermal stability, neutral pH optima, high specificity for phytate and proteolysis resistance (Fu et al. 2008). Some previous reports have suggested that the use of both *Bacillus *and fungal phytases together would be a promising alternative owing to their synergistic activities throughout the animal gastrointestinal tract (Elkhalil et al. 2007). The enormous potential of *Bacillus *phytases has motivated researchers to attempt their overproduction in microbial systems. Because the original strains produce low level of phytases, phytase gene heterologous expression was widely used to improve their production yield. For instance, *Pichia pastoris *has been successfully used as host for heterologous expression of some phytase genes from *Bacillus *(Guerrero-Olazaran et al. 2010). In prokaryotes, except for the expression system used by Tran et al. (2010), which allowed the production of the *Bacillus *sp. MD2 phytase at 327 U ml^-1 ^by fed-batch cultivation, the majority of earlier attempts with expression of *Bacillus *phytases in *Escherichia coli *have resulted in production of inclusion bodies which entails additional steps for recovery of the active enzymes (Rao et al. 2008). As alternative, few expression systems have been developed in *Bacillus subtilis*, a microorganism generally recognized as safe (GRAS) and extensively used to produce in large scale, food-grade enzymes at cost-effective prices thanks to its high ability to secrete soluble and active proteins (Chen et al. 2010). Another advantage of *B. subtilis*, is that domesticated laboratory strains like "168" are naturally competent and even for environmental isolates, competence can be genetically established (Nijland et al. 2010). In general, vectors replicating in a theta (θ) mode known for their segregational and structural stability were used for expression (Chiang et al. 2010) and multimeric plasmid DNA forms were used for transformation (de Vos and Venema 1981). The literature comprises several studies dealing with the production of *Bacillus*-derived phytases in *B. subtilis*. For instance, *B. amyloliquefaciens *DS11 phytase was produced with an activity of 2 U ml^-1 ^(Kim et al. 1999), the PhyC phytase originating from *B. subtilis *VTTE-68013 was overexpressed at 28.7 and 47.7 U ml^-1 ^by Kerovuo et al. (2000) and Vuolanto et al. (2001) respectively, and the *168phyA *and *phyL *encoded phytases were overexpressed at activity levels of 35 and 28 U ml^-1 ^respectively (Tye et al. 2002).

In addition to heterologous expression, overproduction of enzymes by optimization of fermentation conditions can be considered a promising strategy. The use of conventional one-dimensional methods is tedious, time consuming and costly. It also leads to misinterpretation of the results because the interaction between different factors is overlooked. Statistical methods like Plackett-Burman (PB), Box-Behnken (BB) and Central composite (CC) designs that involve a minimum number of experiments for studying several factors, have been employed to improve the production of many enzymes such as α-amylase (Kammoun et al. 2008), xylanase (Fang et al. 2010) and phytase (Kammoun et al. 2011; Singh and Satyanarayana 2008).

We previously characterized the extracellular calcium-dependent phytase from *Bacillus subtilis *US417 (PHY US417) (Farhat et al. 2008). This enzyme exhibiting perfect stability at pH value ranging from 2 to 9 and high thermal stability was optimally active at pH 7.5 and 55°C (Farhat et al. 2008). Considering the high potential of PHY US417 for use as feed supplement, the present investigation deals with the overexpression of the gene encoding this enzyme in *B. subtilis *168 using a transformation protocol involving, as far as we know, for the first time the mutlimerisation of a cloning vector carrying the pAMβ1 replication origin. Furthermore, it also reports a sequential optimization strategy to enhance phytase production by the recombinant *Bacillus *through statistically designed experiments as well as the biochemical characterization of the recombinant phytase in comparison with the native enzyme.

## Materials and methods

### Bacterial strains, plasmids and media

*B. subtilis *168 (*trpC2*) and *E. coli *DH5α respectively used as hosts for expression of plasmid-encoded phytase and molecular cloning were generously gifted by Dr. Emmanuelle Maguin. pMSP3535 (Bryan et al. 2000) was the cloning vector for phytase overexpression. This shuttle vector carries the replication origin of the *Enterococcus faecalis *pAMβ1 plasmid replicating by a θ mechanism in a broad range of Gram-positive bacteria and showing high segregational stability. *E. coli *and *B. subtilis *have been grown in Lauria-Bertani (LB) medium. When needed, erythromycin has been added at 160 and 5 μg ml^-1 ^for *E. coli *and *B*. *subtilis *respectively.

### Substrates and chemicals

Phytic acid sodium salt hydrate from rice (P0109) was purchased from Sigma. Yeast extract (64343) and ammonium sulphate (ADB0060) were acquired from Biorad and Bio Basic Inc. respectively. Wheat bran was obtained from the local company "Nutrisud/Medimix". All other chemicals used in this study are commercially available in analytical grade.

### DNA manipulation

General molecular biology techniques were performed as described by Sambrook et al. (1989). DNA restriction and modification enzymes were used according to the supplier's recommendations. PCR amplifications were carried out using *Pfu *DNA polymerase from BIOTOOLS (Madrid-Spain).

### Construction of phytase overexpression plasmid

To overproduce PHY US417 in *B. subtilis *168, a 1311 bp *Sph*I-*Sal*I DNA fragment from the pAF2 plasmid (Farhat et al. 2008) carrying the whole *phy *US417 gene was sub-cloned in pMSP3535 linearized by *Sph*I-*Xho*I to produce pAF3 (9638 bp).

### *Bacillus subtilis *transformation

*B. subtilis *was transformed according to the method of Anagnostopoulos and Spizizen (1961) with some modifications. To obtain naturally competent cells, *B. subtilis *168 was grown in the Spizizen minimal medium (SMM): 80 mM K_2_HPO_4_, 45 mM KH_2_PO_4_, 15 mM (NH_4_)_2_SO_4 _and 3.8 mM Na_3_-citrate, supplemented with 5 mM MgSO_4_, 5 g l^-1 ^glucose, 0.5 g l^-1 ^tryptophan and 0.1 g l^-1 ^casaminohydrolysate. For efficient DNA uptake of pAF3 and pMSP3535 (negative control) by *B. subtilis*, the plasmid DNA (1 μg) was linearized by *Nsi*I and self-ligated *in vitro *to generate multimeric plasmidic forms. After dilution of competent cells (10^-1^) in SMM containing 20 mM MgCl_2 _and 5 g l^-1 ^glucose, pAF3 or pMSP3535 plasmid DNA multimers were added, and the samples were incubated for 20 min at 37°C. Transformation mixtures were subsequently spread on LB agar containing erythromycin (5 μg ml^-1^). *B. subtilis *transformants were screened for the ability to produce phytase activity on LB agar supplemented with phytic acid (3 mM) by using the well-known two step counterstaining treatment (Bae et al. 1999). Colonies surrounded by clear zones were tested by PCR to confirm the presence of the *phy *US417 gene.

### Phytase production by submerged fermentation

Prior to optimization, a liquid basal medium (LBM) that contained 50 g l^-1 ^wheat bran; 0.4 g l^-1 ^(NH_4_)_2 _SO_4_; 0. 2 g l^-1 ^Mg SO_4 _7 H_2_O and 2.2 g l^-1 ^CaCl_2 _at pH 6.5, was used for phytase production by *B. subtilis *168 carrying pAF3. Cultures were carried out in 500 ml flasks containing 100 ml of medium, inoculated at 0.1 OD_600 _from 19 h-old culture grown on LB and incubated at 37°C for 72 h under shaking speed of 250 rpm. After cultivation, the culture broth was centrifuged at 10000 rpm for 10 min and the cell-free supernatant was used for the determination of phytase activity.

### Assays for phytase activity

Phytase activity assays were carried out at 65°C for 30 min (for rPHY US417) as described by Farhat et al. (2008). For the reference, the color-stop mix was added prior to the phytic acid solution and the reaction mixture was not incubated at 65°C (kept at room temperature). One phytase unit (U) was defined as the amount of enzyme capable of releasing 1 μmol of inorganic phosphate (Pi) min^-1 ^(from phytic acid) under the optimal conditions.

### Identification of critical culture variables using Plackett-Burman design

For a screening purpose, various medium components and culture parameters were evaluated. Using a Plackett-Burman (PB) factorial design, each factor was examined in two coded levels: -1 and +1 respectively for low and high level. Table 1 shows the 15 assigned variables under investigation as well as levels of each variable used in the experimental design, whereas Table 2 illustrates the design matrix (16 trials). All experiments were carried out in triplicate and the average of the phytase activity was taken as response (Table 2).

**Table 1:** Assigned concentrations of different parameters and their levels in Plackett-Burman design for phytase production

Codes	Factors	Level (-1)	Level (+1)
A	T	30	37
B	pH	5.5	7.5
C	Shaking speed (rpm)	150	250
D	Inoculum size (OD600_f_)	0.05	0.5
E	KH_2_PO_4_	0	5
F	Methanol	0	5
G	Glycerol	0	5
H	Galactose	0	5
I	Urea	0	5
J	Casein hydrolysate	0	5
K	Yeast extract	0	5
L	(NH_4_)_2 _SO_4_	0	5
M	Triton X-100	0	5
N	Phytic acid	0	0.01
O	Corn steep liquor	0	10

**Table 2:** Plackett-Burman design for 15 variables with coded values along with the observed results for phytase production

Trial	A	B	C	D	E	F	G	H	I	J	K	L	M	N	O	Phytase activity (U ml^-1^)
1	+	-	-	-	+	-	-	+	+	-	+	-	+	+	+	3.96 ± 0.01
2	+	+	-	-	-	+	-	-	+	+	-	+	-	+	+	13.39 ± 0.34
3	+	+	+	-	-	-	+	-	-	+	+	-	+	-	+	27.20 ± 0.62
4	+	+	+	+	-	-	-	+	-	-	+	+	-	+	-	28.18 ± 0.70
5	-	+	+	+	+	-	-	-	+	-	-	+	+	-	+	24.58 ± 0.53
6	+	-	+	+	+	+	-	-	-	+	-	-	+	+	-	7.52 ± 0.18
7	-	+	-	+	+	+	+	-	-	-	+	-	-	+	+	11.68 ± 0.30
8	+	-	+	-	+	+	+	+	-	-	-	+	-	-	+	23.60 ± 0.59
9	+	+	-	+	-	+	+	+	+	-	-	-	+	-	-	10.80 ± 0.27
10	-	+	+	-	+	-	+	+	+	+	-	-	-	+	-	9.84 ± 0.25
11	-	-	+	+	-	+	-	+	+	+	+	-	-	-	+	26.01 ± 0.65
12	+	-	-	+	+	-	+	-	+	+	+	+	-	-	-	10.68 ± 0.26
13	-	+	-	-	+	+	-	+	-	+	+	+	+	-	-	7.45 ± 0.19
14	-	-	+	-	-	+	+	-	+	-	+	+	+	+	-	26.64 ± 0.62
15	-	-	-	+	-	-	+	+	-	+	-	+	+	+	+	0.94 ± 0.02
16	-	-	-	-	-	-	-	-	-	-	-	-	-	-	-	11.17 ± 0.40
E(Xi)	0.88	2.83	12..94	-0.36	-5.63	1.32	-0.11	-2.76	1.02	-4.70	5.00	3.41	-3.18	-4.92	2.38	

The contrast coefficient (*E_(Xi)_*) of each examined factor, the standard error (*SE*) of the concentration effect and the significant level (p-value) of the effect of each concentration were determined as described by Kammoun et al. (2011).

### Box-Behnken Design

To establish the response surface in the experimental region and to identify the optimum conditions for enzyme production, a Box-Behnken (BB) design was applied. Table 3 presents the design matrix, consisting of 13 trials to study the 3 most significant variables affecting phytase activity, which have been selected using the PB design [shaking speed in rpm (N), concentration (g l^-1^) of yeast extract (YE) and of ammonium sulphate (AS)]. Each variable was studied on three levels, coded -1, 0, and +1 respectively for low, middle, and high values. The prediction of optimum independent variables was identified by fitting the experimental data using second order polynomial regression equation including individual and cross effect of each variable as described by Kammoun et al. (2011).

**Table 3:** Box Behnken factorial experimental design representing response of phytase activity (U ml^-1^) as influenced by shaking speed and concentration of yeast extract and ammonium sulphate

Exp	X1	X2	X3	Phytase activity (U ml^-1^)	Phytase activity predicted (U ml^-1^)
1	300	5	10	35.73 ± 0.17	35.18
2	300	15	10	42.92 ± 0.21	43.36
3	250	5	5	30.15 ± 0.15	29.86
4	250	5	15	33.10 ± 0.13	34.43
5	250	15	5	34.51 ± 0.2	32.79
6	250	15	15	35.79 ± 0.17	37.37
7	200	10	5	17.51 ± 0.08	18.75
8	300	10	5	38.62 ± 0.19	39.89
9	200	10	15	24.57 ± 0.12	23.33
10	300	10	15	45.63 ± 0.36	44.47
11	250	10	10	34.36 ± 0.17	36.52
12	250	10	10	34.93 ± 0.10	36.52
13	250	10	10	41.17 ± 0.23	36.52

### Validation of the experimental model and scale up in laboratory fermenter

Fermentation for phytase production under the optimized conditions predicted by the model was carried out at 300 rpm in the presence of 12.5 and 15 g l^-1 ^of YE and AS respectively. Supernatant samples were taken at regular intervals by centrifugation and assayed for phytase activity. *Bacillus *cell density (10^8 ^CFU ml^-1^) was monitored during growth by preparing serial decimal dilutions and plating on LB agar supplemented with 5 μg ml^-1 ^of erythromycin. Plates were incubated overnight at 37°C and the resulting colony forming units (CFU) were counted. After validation of the model in flasks, assays of batch fermentation were performed in a 7 l Infors HT fermenter (Infors AG, Rittergasse 27, 4013 Bottmingen, Switzerland) with a working volume of 3.5 l under the optimized culture conditions. The fermenter was operated at 37°C, 500 rev min^-1^, 1 vvm of aeration and with pH control at 7.5. The cells were harvested at different time periods (6, 24, 30, 42, 48, 60 and 72 h post inoculation) and the cell-free supernatants were used to determine the phytase activity.

### Software tools

The statistical software package "SPSS" (Version 11.0.1 2001, LEAD Technologies, Inc., USA) was used to analyze the experimental data and EXCEL software (Version 2003, Microsoft office, Inc., USA) was used to generate the response surface that allow to find out the levels of the variables for maximal phytase activity.

### Purification, identification and characterization of the recombinant phytase

rPHY US417 was produced after cultivation of the recombinant *Bacillus *under the optimized fermentation conditions for 72 h at 37°C. The enzyme was then purified as described by Farhat et al. (2008) and its purity was estimated using sodium dodecyl sulphate polyacrylamide gel electrophoresis (SDS-PAGE) and Coomassie blue staining as described by Laemmli (1970). Electrophoresis was carried out on a 10% polyacrylamide gel at room temperature at a constant voltage of 150 V for one hour. To confirm that the purified protein corresponds to the phytase being cloned, we have performed enzyme digestion with trypsin, and the obtained peptide mixtures were analyzed using a Voyager DE STR MALDI-TOF mass spectrometer (Applied Biosystems) as described by Rhimi et al. (2011). Recorded MS/MS spectra were compared to theoretical fragmentations of a trypsinolysed PHY US417 protein (GenBank accession no. CAM58513). Automated Edman's degradation was performed as described in Farhat et al. (2008) to determine the first amino acid of the mature rPHY US417.

The temperature profile of rPHY US417 was obtained by determining its activity between 37 and 80°C at pH 7.5. Thermostability was checked by incubating the enzyme up to 1 h at 75°C in 0.1 M Tris-HCl buffer pH 7.5 supplemented with 5 mM CaCl_2_. For control heat treatment experiments (without addition of calcium), the enzyme solution was dialyzed against 0.1 M Tris-HCl buffer pH 7.5 and heating was performed in this buffer in the presence of 2 mM ethylenediaminetetraacetic acid (EDTA). At certain time intervals, samples were withdrawn and the residual activity was measured right after heat treatment. The effect of pH (from 3 to 9.5) on rPHY US417 activity was investigated at 65°C using the same buffer solutions reported in Farhat et al. (2008). The effect of pH on rPHY US417 stability was performed by incubating the enzyme at pH ranging from 2 to 9 for 1 h at 37°C, followed by measuring its residual activity. For comparison, similar assays with PHY US417 purified from *B. subtilis *US417 were performed under the enzyme optimal conditions (Farhat et al. 2008).

## Results

### Cloning of *phy *US417 gene into pMSP3535 and expression of recombinant phytase in *B. subtilis *168

**Figure 1:**
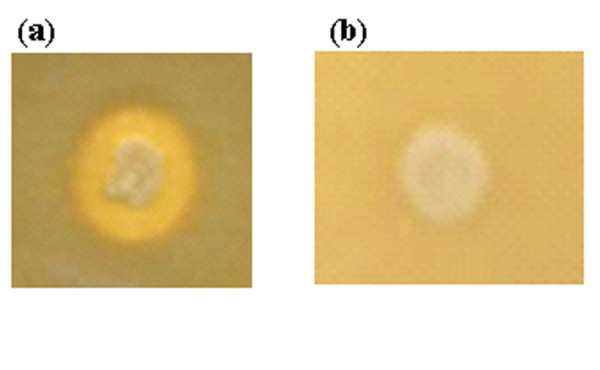
**Detection of phytase activity in *Bacillus subtilis *168 transformants**. Colonies of *B. subtilis *168 carrying pAF3 (a) and pMSP3535 (b) were grown on LB agar supplemented with 3 mM of phytic acid for 24 h and then phytase activity has been visualized by the two step counterstaining treatment.

A plasmid construct (pAF3) in which the *phy *US417 gene (with its native promoter) was cloned in pMSP3535 was prepared in *E. coli*. Then, for efficient DNA uptake by naturally competent *B. subtilis *168 cells, multimers of pAF3 and pMSP3535 were constructed *in vitro *and used for transformation (for the first time for vectors carrying the replication origin of the *E. faecalis *pAMβ1 plasmid). Erythromycin resistant colonies of *B. subtilis *transformed with pAF3 but not those with pMSP3535, showed clear zones of phytic acid hydrolysis around (Figure 1). This was correlated with the detection by PCR of the presence of the *phy *US417 gene. In liquid basal medium (LBM), maximum extracellular phytase activity of 3.5 U ml^-1 ^was obtained after cultivation of *B. subtilis *168 carrying pAF3 for 72 h at 37°C. This was 5.5 fold higher than the phytase yield achieved by the native *B. subtilis *US417 strain under original conditions (Farhat et al. 2008).

In order to assess the stability of the maintenance of pAF3 in *B. subtilis *168, cultures of the recombinant *Bacillus *inoculated from starter cultures (made under selective pressure) were grown for 72 h at 37°C with and without antibiotic selection. No decrease in phytase secretion was detected under nonselective conditions. Even after inoculation of fresh medium and another round of growth, no differences were obvious and the totality of *Bacillus *cells are harbouring the antibiotic marker in late fermentation as revealed by plate counting.

### Evaluation of culture conditions affecting phytase production by the recombinant *Bacillus*

The factors affecting recombinant phytase (rPHY US417) production by *B. subtilis *168 carrying pAF3 were identified using a PB statistical design. Settings of 15 independent variables were examined, as shown in Table 1. The experiments were carried out according to the experimental matrix presented in Table 2 where the phytase activity (U ml^-1^) was the measured response. A wide variation of phytase yield from 0.94 to 28.18 U ml^-1 ^was found among the 16 trials, as shown in Table 2 thereby emphasizing the importance of the screening step to identify the most influent variables. The analysis of the contrast coefficient (*E _(Xi)_*) has shown that the shaking speed (N) and the concentration (g l^-1^) of yeast extract (YE) and ammonium sulphate (AS) have pronounced influence on phytase production with *E _(Xi) _*varying between 3.41 and 12.94 (Table 2). For the remaining parameters, those with a positive *E _(Xi) _*(enhance the phytase production) like T, pH, methanol, urea and corn steep liquor were maintained in RSM experiments at their high levels. However, the variables that possess a negative value of *E _(Xi) _*were eliminated, except for the inoculum size (indispensable) which was preserved at its lower level.

### Response surface methodology for optimization of phytase production

The response surface methodology (RSM) was widely applied to optimize phytase production by several microorganisms (Kammoun et al. 2011; Singh and Satyanarayana 2008; Singh and Satyanarayana 2006). Thus, to determine the optimum response region for phytase activity, the significant independent variables which are N (X1), concentration (g l^-1^) of YE (X2) and AS (X3) were further studied at three levels: -1, 0, and +1. The 13-trial design matrix illustrating the BB design is represented in Table 3 along with the predicted and observed phytase activity.

The regression equation obtained after the analysis of variance (ANOVA) provided the level of enzyme production as a function of the shaking speed (N) and the concentration (g l^-1^) of YE and AS (Table 4). The phytase activity (U ml^-1^) could be predicted by the following equation:

**Table 4:** Analysis of the main variables affecting phytase production by Student's test

	Non standardized Coefficients		Standardized Coefficients	*t*	Significance
	**B**	**Standard Error**	**Bêta**		
Coefficients	-131.98	37.153		-3.552	0.009
x_1_	1.09	0.294	5.024	3.694	0.007
x_3_	0.46	1.744	0.250	2.621	0.034
x_1_^2^	-0.002	0	-4.630	-3.314	0.013
x_2_^2^	-0.12	0,053	-1.121	-2.192	0.064
x_1_x_2_	0.10	0.004	1.415	2.606	0.035

$$\text{Y}=-131.98+1.09*\text{N}+0.46*\text{AS}-0.002*{\left(\text{N}\right)}^{2}-0.12*{\left(\text{YE}\right)}^{2}+0.10*\text{N}*\text{YE}$$

Where Y is the phytase activity (U ml^-1^), N the shaking speed (rpm), YE and AS are the concentration (g l^-1^) of YE and AS respectively.

This equation means that the phytase production is affected by the parameters shaking speed (N, N^2^), ammonium sulfate (AS), yeast extract (YE^2^) and the interaction between N and YE. The significance levels of the coefficients were determined by the Student's test which allows not only identification of the parameters that have significant effect on phytase production but also the level of this effect. From Table 4 the effects of N, AS and the interaction between N and YE were found to be significant (p < 0.05).

For the above equation, the multiple correlation coefficient (R) and the determination coefficient (R^2^) are used to evaluate the validity of the model. In this trial, the value of R was 0.97, which reflects the high degree of correlation between the experimental and predicted values of phytase activity. Pertaining to R^2 ^that is indicative of model fitting, its value was 0.94 which means that 6% of the total variations were not explained by the model. The value of the adjusted determination coefficient (adj. R^2^) was calculated to be 0.89, which indicates a high significance of the model. Together, the determined coefficients indicate an excellent adequacy of the model to the experimental data.

**Figure 2:**
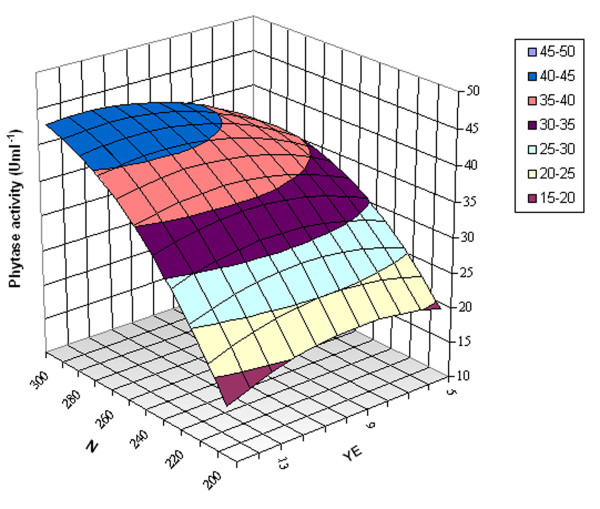
**Response surface plot for phytase activity (U ml^-1^) observed as a response to the interaction of N and concentration (g l^-1^) of YE as variables and concentration (g l^-1^) of AS at central point**.

The response surface (3D) plot for phytase activity was generated for two factors [N and concentration (g l^-1^) of YE] while the concentration of AS was kept constant (15 g l^-1^). Figure 2 illustrates the quite significant interaction between N and the concentration of YE. This was confirmed by the low value of P (0.035) as mentioned in Table 4. The phytase activity increases significantly with increasing the shaking speed specially for high YE concentrations (Figure 2). The RSM plot also shows that the maximum response is in the shape of a small area limited by values of N in the range of 290-300 rpm and concentrations of YE varying from 10 to 12.5 g l^-1 ^(Figure 2). The predicted maximum phytase activity of 45.63 U ml^-1 ^can be reached using a shaking speed of 300 rpm in the presence of respectively 12.5 and 15 g l^-1 ^of YE and AS.

### Optimum validation and scale up in laboratory fermenter

**Figure 3:**
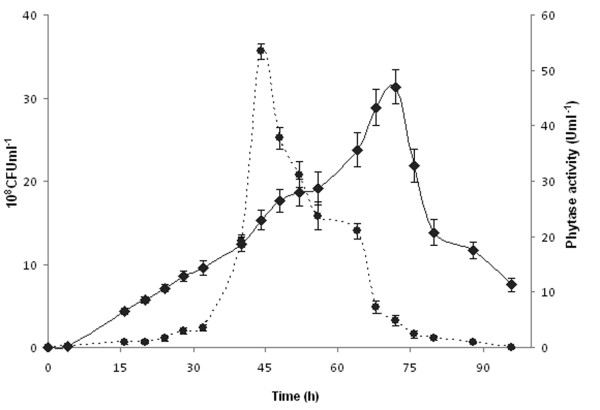
**Extracellular phytase activity (U ml^-1^) (black diamond) and growth (10^8 ^CFU ml^-1^) (black circle) of the *Bacillus subtilis *168 strain carrying pAF3 during cultivation under the optimized conditions predicted by the model**. Values represent the means of triplicate experiments with comparable results.

For the validation of the model predicting phytase activity, kinetics of bacterial growth and phytase activity were investigated experimentally by applying the conditions allowing the achievement of the predicted maximum phytase activity of 45.63 U ml^-1 ^(shaking speed of 300 rpm in the presence of 12.5 g l^-1 ^of YE and 15 g l^-1 ^of AS). After a short Lag phase of about 5 h, we witness an exponential phase of bacterial growth and maximum number of viable cells was attained after a period of 45 h (Figure 3). This exponential growth was accompanied with a rapid increase in phytase activity. From 45 h, growth ceases (entry to stationary phase) and we assist to a decline phase (death phase) that was may be accentuated by the high ATPase activity of the produced US417 phytase as previously demonstrated for the native PHY US417 enzyme purified from the *B. subtilis *US417 strain (Farhat et al. 2008). Despite this decline in growth, phytase activity continues to increase reaching its maximum level of 47 U ml^-1 ^after a growing period of 72 h. This temporal difference between maximal growth and phytase activity can be explained in part by the time needed for complete functional recognition and processing of the signal peptide of the phytase precursor by the secretion machinery of *B. subtilis *168. Our results show a nearly perfect agreement between the predicted and experimental responses. It is worth noting that applying the RSM allowed to reach a phytase activity level which was about 13.4 and 1.66 fold higher than that obtained without optimization (3.5 U ml^-1^) and following the critical variables screening study (28.2 U ml^-1^) respectively.

After optimum validation under shake flask conditions, batch cultivation was performed in laboratory scale fermenter of 7 l capacity. This trial resulted in the sustainable production of rPHY US417 since a maximum phytase titer of about 45 U ml^-1 ^was reached after 42 h of cultivation.

### Functional characterization of the recombinant phytase

**Figure 4:**
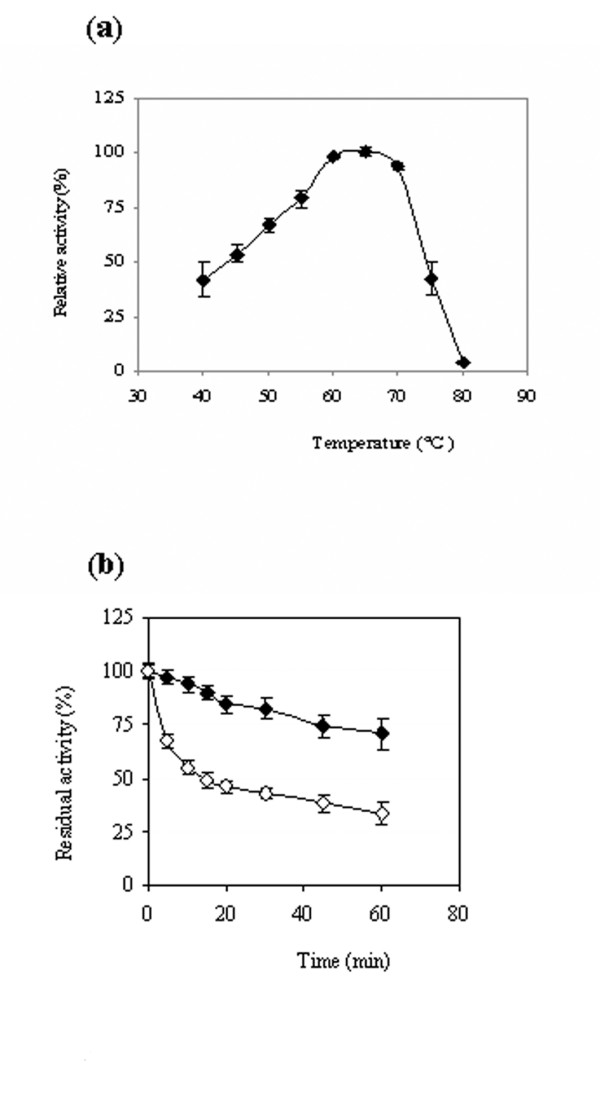
**Effect of temperature on rPHY US417 activity and stability**. (a) Temperature profile of rPHY US417. (b) Thermal stability of rPHY US417 at 75°C in the absence and presence of calcium are represented by white and black diamonds respectively.

The mature rPHY US417 was purified as described by Farhat et al. (2008) and its identity was confirmed by mass spectrometry. It possesses a specific activity of 30.9 U mg^-1 ^and a molecular mass of 41 kDa like the native PHY US417 phytase produced by *B. subtilis *US417, as revealed by SDS-PAGE analysis (data not shown). N-terminal sequencing confirmed that the first amino acid of rPHY US417 is leucine 30 as the native enzyme (Farhat et al. 2008). The purified rPHY US417 showed also dependence toward calcium for its catalytic activity. Increasing the concentration of calcium enhanced the enzyme activity which reaches its highest level in the presence of 1 mM CaCl_2 _like the native PHY US417 enzyme (data not shown). Investigation of the effect of pH on rPHY US417 activity and stability, showed that similar to the native phytase, this enzyme was optimally active at neutral pH range with the highest activity at pH 7.5 and perfectly stable at pH value ranging from 3 to 9 (data not shown). On the contrary and for unknown reasons, the study of the effect of temperature on enzyme activity and thermal stability, illustrated that rPHY US417 exhibited an improved thermoactivity and thermostability compared to PHY US417. Indeed, it was optimally active at 65°C (instead of 55°C) and recovered about 90 and 55% of its activity (77 and 0% for the native enzyme) after heating for 10 min at 75°C in the presence and absence of 5 mM CaCl_2 _respectively (Figure 4ab).

## Discussion

In the present study, we have developed an efficient system for cost-effective large-scale production of the thermostable PHY US417 phytase from *B. subtilis *US417 in *B. subtilis *168. Accordingly, the phytase-encoding gene was cloned in the pMSP3535 vector, and then, for the first time for a plasmid carrying the pAMβ1 replication origin, multimers of the resulting pAF3 plasmid were used to transform *Bacillus*. The stability of the maintenance of pAF3 in the recombinant *Bacillus *strain even under nonselective growth conditions was proved. This observation is consistent with previous findings showing that the θ-type replication mode confers higher segregational and structural stability of plasmids in *Bacillus *compared to the rolling-circle-type replication (Bron and Luxen 1985).

Subsequently, to enhance the level of phytase production by the recombinant *Bacillus*, we applied an optimization strategy based on statistical designs. Among the parameters screened by PB design, the shaking speed (N) and the concentration (g l^-1^) of yeast extract (YE) and ammonium sulphate (AS) were selected based on their highly significant positive effect on phytase production. The great influence of shaking speed has also been reported for the production of the rLlALP2 phytase from lily pollen in *Pichia pastori*s and the phytase from *Aspergillus niger *(Johnson et al. 2010;Papagianni et al. 2001). Concerning the nitrogen sources, the concentrations of YE and AS have also been identified as critical variables that enhance phytase production as in *A. niger *van Teigham (Vats et al. 2004) and *Sporotrichum thermophile *(Singh and Satyanarayana 2008) respectively. In contrast, peptone, sodium nitrate and urea have been reported to be the preferred nitrogenous sources for phytase production by respectively *A. niger *CFR335 (Gunashree and Venkateswaran 2008), *A. niger *NCIM 563 (Soni and Khire 2007) and *P. anomala *(Kaur and Satyanarayana 2005).

In addition to the illustration of the vital role of nitrogen sources for the enzyme synthesis, the results of our study showed that supplementation of phosphate in the culture medium in the form KH_2_PO_4_, even at low concentration, repressed the enzyme production (Table 2) as observed by Singh and Satyanarayana (2006) for phytase production by *S. thermophile*. In general, previous reports have demonstrated that phytase expression in Pi-limiting conditions is much higher than that under Pi sufficiency (Kammoun et al. 2011;Singh and Satyanarayana 2008;Singh and Satyanarayana 2006). The poor phytase production in some media might be related to the abundance of Pi in the ingredients of these medias like wheat bran and YE (Servi et al. 2008; Singh and Satyanarayana 2006;Vuolanto et al. 2001) and thus, external addition of phosphate could accentuate the repression of enzyme synthesis as suggested by Kammoun et al. (2011).

Following the critical variables selection, the RSM was applied to further optimize the enzyme production. This allowed reaching a maximum phytase activity of 47 U ml^-1^, which represents 73 fold higher than the activity produced by the native US417 strain (Farhat et al. 2008). In laboratory fermenter, the scale up experiments performed are promising, and the enzyme titer is expected to increase after further optimization of the fermentation parameters like the aeration, inoculum size and agitation speed. The use of feed-back cultivation strategy known to prevent nutrient limitation might provide higher cell density and significant increase in the phytase yield as observed by Tran et al. (2010).

In conclusion, thanks to heterologous expression of the phytase gene from *B. subtilis *US417 in *B. subtilis *168 using the new efficient expression system developed and applying experimental designs optimization, we succeeded to reach maximum phytase yield of 47 U ml^-1 ^which represents one of the highest phytase activity achieved so far in *Bacillus*. The findings obtained for phytase production in this study suggest that future application of the expression strategy developed herein for overproduction of recombinant proteins in *Bacillus *is highly promising.

## Competing interests

The authors declare that they have no competing interests.
